# Suppression of Gelatinase Activity in Human Peripheral Blood Mononuclear Cells by Verapamil

**Published:** 2014-02-03

**Authors:** Fatemeh Hajighasemi, Neda Kakadezfuli

**Affiliations:** Department of Immunology, Faculty of Medicine, Shahed University, Tehran, Iran

**Keywords:** Verapamil, Gelatinase, Mononuclear Cells

## Abstract

**Objective:**

Gelatinases are a large group of proteolytic enzymes that belong to the matrix metalloproteinases (MMPs). MMPs are a broad family of peptidases, which proteolyse the extracellular matrix and have an important role in inflammation. Verapamil is a calcium channel blocker extensively used in the treatment of numerous cardiovascular diseases such as arrhythmia and hypertension. The anti-tumor and anti-inflammatory effects of verapamil have also been shown. In this study, the effect of verapamil on gelatinase activity in human peripheral blood mononuclear cells (PBMCs) has been assessed *in vitro*.

**Materials and Methods:**

In this experimental study, PBMCs from healthy adult volunteers were isolated by ficoll-hypaque-gradient centrifugation. The cells were then cultured in complete RPMI-1640 medium and after that incubated with different concentrations of verapamil (0–200 μM) in the presence or absence of phytoheamagglutinin (PHA) (10 μg/ml) for 48 hours. The gelatinase A (MMP-2)/gelatinase B (MMP-9) activity in cell-conditioned media was then evaluated by gelatin zymography. Statistical comparisons between groups were made by analysis of variance (ANOVA).

**Results:**

Verapamil significantly decreased the MMP-2/MMP-9 activity in human PBMCs after 48 hours incubation time compared with untreated control cells. The association was dose-dependent.

**Conclusion:**

In this study verapamil exhibited a dose-dependent inhibitory effect on gelatinase A and gelatinase B activity in human PBMCs. It seems that the anti-inflammatory properties of verapamil may be in part due to its inhibitory effects on gelatinase activity. Regarding the beneficial effects of MMPs- inhibitors in the treatment of some cardiovascular diseases, the positive effect of verapamil on such diseases may be in part due to its anti-MMP activity. Verapamil with its inhibitory effects on gelatinases activity may be a useful MMP-inhibitor. Given the beneficial effect of MMP-inhibitors in some cancerous, inflammatory and autoimmune disorders, it seems likely that verapamil could also be used to treat these diseases.

## Introduction

Gelatinases are a family of proteolytic enzymes belonging to the matrix metalloproteinases (MMPs) (1). MMPs are a group of zinc-dependent enzymes, which proteolyse the extracellular matrix (2, 3). Gelatinases degrade collagen type ІV and V and are divided into gelatinase A (MMP-2) and gelatinase B (MMP-9) with molecular weights of 72 and 92 KD, respectively (4, 5). Gelatinases play an important role in inflammation, autoimmunity, cancer progression and metastasis (6-9). Verapamil is a calcium (Ca2+) channel blocker extensively used in the treatment of numerous cardiovascular diseases, such as arrhythmia and hypertension (10, 11). Anti-tumor and antiinflammatory effects of verapamil have also been shown (12, 13). The inhibitory effect of verapamil on lipopolysaccharide (LPS)-induced pro-inflammatory cytokine production and NF-κβ activation has been shown *in vivo* (14, 15), and the therapeutic properties of a Ca2+ channel blocker in inflammatory bowel disease have been described (16). In addition, down regulation of proinflammatory factors such as superoxide and nitric oxide (NO) by verapamil through a Ca2+ channel-independent pathway (13) and the inhibitory effect of verapamil on MMP-9 activity in murine mammary tumor cells has been reported (12).

Mononuclear cells play an important role in inflammation (17, 18) through several mechanisms such as regulating the extracellular turnover. This occurs via the production of a number of mediators such as inflammatory cytokines and MMPs (19-21). Production of gelatinases by peripheral blood mononuclear cells (PBMCs) has also been shown (22). Given the anti-inflammatory effects of verapamil and the important role of mononuclear cells and MMPs in inflammation, in this study we assessed the effect of verapamil on gelatinase (MMP-2 and MMP-9) activity in human PBMCs.

## Materials and Methods

This experimental study was approved by The Deputy Director of Research in the Faculty of Medicine at Shahed University.

### Reagents

RPMI-1640 medium, penicillin, streptomycin, PHA (phytoheamagglutinin) and trypan blue (TB) were obtained from Sigma (USA). MTT (3-[4,^5^-dimethyl thiazol-2,^5^-diphenyltetrazolium bromide]) was purchased from Merck (Germany). Fetal calf serum (FCS) was from Gibco (USA). Verapamil was purchased from Sobhandarou Pvt. Co. Ltd (Tehran, Iran). Microtiter plates, flasks and tubes were from Nunc (Falcon, USA).

### Preparation of verapamil

Verapamil was dissolved in distilled water and stored as a stock at -20˚C until use. The stock was diluted in culture medium in order to prepare appropriate concentrations before use.

### Peripheral blood mononuclear cells isolation

PBMCs from the venous blood of healthy adult volunteers were isolated by ficoll-hypaque-gradient centrifugation. Subsequently, the cells were washed three times in phosphate buffer saline (PBS). The cells were then resuspended in RPMI- 1640 medium supplemented with 10% FCS and were incubated in 5% CO_2_ at 37˚C.

### Cell culture and treatment

The method used for cell culture and treatment has been described in detail previously (23). Briefly, human PBMCs were cultured in RPMI- 1640 medium supplemented with 10% FCS, penicillin (100 IU/ml) and streptomycin (100 μg/ml) at 37˚C in 5% CO_2_. The cells were seeded at a density of 1×106 cells/ml and then treated with different concentrations of Verapamil (0- 200 μM) in the presence of PHA (10 μg/ml) for 48 hours. Afterward the supernatants from the cell cultures were collected, centrifuged and stored at -20˚C for subsequent tests. All experiments were done in triplicate.

### Evaluation of MMP-2 and MMP-9 activity by gelatin zymography

MMP-2 and MMP-9 activity in cell-conditioned media were evaluated using the gelatin zymography technique according to the modified Kleiner and Stetler-Stevenson method (1994, 24) as previously described (25). Briefly, cell culture supernatants were subjected to SDS-PAGE on 10% polyacrylamide gel copolymerized with 2 mg/ ml gelatin in the presence of 0.1% SDS under non-reducing conditions at a constant voltage of 80 V for three hours. After electrophoresis,the gels were washed in 2.5% Triton X-100 for one hour to remove the SDS and then incubated in a buffer containing 0.1 M Tris-HCl, pH=7.4 and 10 mM CaCl2 at 37˚C overnight. Afterwards, the gels were stained with 0.5% Coomassie brilliant blue (Coomassie blue dissolved in 40% ethanol, 10% acetic acid) for 1 hour and then destained. Proteolytic enzyme activity was detected as clear bands against a blue background indicating lysis of gelatin. The supernatants from serum-free cultured HT1080 cells obtained from NCBI (National Cell Bank of Iran, Pasteur Institute of Iran, Tehran) were used as a molecular weight marker for MMP-2 and MMP-9 as described before (26).

The relative intensity of the gelatin lysis bands compared to the control was measured using UVI Pro gel documentation system (Vilber Lourmat, Marne-la-Vallee Cedex 1, France) and expressed as relative gelatinolytic activity.

### Statistical analysis

MMP-2 and MMP-9 activity measurement in cellconditioned media was performed in three independent experiments and the results were expressed as mean ± SEM. Statistical comparisons between groups were made by analysis of variance (ANOVA). P<0.05 was considered significant. Multiple comparisons were tested using the Tukey method (5%) for statistically significant differences. The software SPSS 11.5 and Excel 2003 were used for statistical analysis and graph making respectively.

## Results

**Fig 1:**
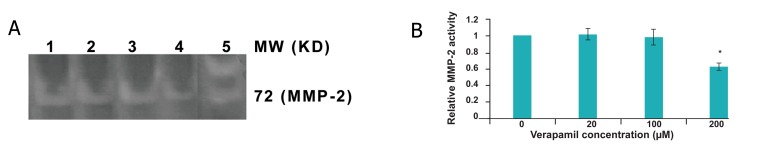
Effect of verapamil on MMP-2 activity in PHA-stimulated human PBMCs. The human PBMCs (1×106 cells/ml), were treated with different concentrations of verapamil (0-200 μM) in the presence of PHA (10 μg/ml) for 48 hours. At the end of treatment, MMP-2 activity in conditioned medium was measured by gelatin zymography. A. Zymogram of MMP-2 activity in human PBMCs treated with verapamil. Lane 1 represents untreated human PBMCs. Lanes 2 to 4 represent verapamil at 20, 100 and 200 μM concentrations respectively. Lane 5 represents the control. B. MMP-2 activity in human PBMCs, was measured by scanning the zymograms and by densitometric analysis of the MMP-2 bands. Data are represented as the mean ± SEM of the three independent experiments. *; P<0.05 was considered significant.

**Fig 2:**
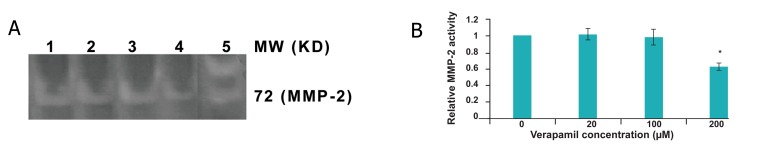
Effect of verapamil on MMP-9 activity in PHA-stimulated human PBMCs. Human PBMCs (1×106 cells/ml), were treated with different concentrations of verapamil (0-200 μM) in the presence of PHA (10 μg/ml) for 48 hours. At the end of treatment, MMP-9 activity in the conditioned medium was measured by gelatin zymography. A. Zymogram of MMP-9 activity in human PBMCs treated with verapamil. Lane 1 represents untreated human PBMCs. Lanes 2 to 4 represent verapamil at 20, 100 and 200 μM concentrations respectively. Lane 5 represents the control. B. MMP-9 activity in human PBMCs, was measured by scanning the zymograms and by densitometric analysis of the MMP-9 bands. Data are mean ± SEM of three independent experiments. *; P<0.05 was considered significant.

Effect of verapamil on gelatinase-A (MMP-2) and gelatinase-B (MMP-9) activity in human PBMCs in different concentrations are shown in figures 1 (A, B) and 2 (A, B).

### Verapamil effect on gelatinase-A (MMP-2) activity in PHA-stimulated human PBMCs

Verapamil significantly decreased the gelatinase- A (MMP-2) activity in PHA-stimulated human PBMCs in a dose-dependent fashion after 48 hours incubation compared with untreated control cells (Fig 1A, B). The decrease in gelatinase- A activity was shown at 200 μM concentration of verapamil.

### Verapamil effect on gelatinase-B (MMP-9) activity in PHA-stimulated human PBMCs

Verapamil significantly decreased the gelatinase- B (MMP-9) activity in PHA-stimulated human PBMCs in a dose-dependent fashion after 48 hours incubation time compared with untreated control cells (Fig 2A, B). The decrease in gelatinase-B activity was seen at a verapamil concentration of 200 μM.

## Discussion

In this study, a 200 μM concentration of verapamil inhibited the gelatinase A (MMP-2) and gelatinase B (MMP-9) activity in human PBMCs. These results are consistent with the study by Farías, et al. (12) in which verapamil suppressed the MMP-9 activity in murine mammary tumor cells. In the Farías, et al. study a 50 μM concentration of verapamil decreased the MMP-9 activity, while in our study the decrease in MMP-9 activity was seen at 200 μM. The discrepancy between our results and those of Farías, et al. may in part be due to the type and origin of the cells used. The cells used by Farías et al. were mouse mammary tumor cells while we used human PBMCs which are normal cells. Thus it seems that verapamil exerts an inhibitory effect on MMP activity in tumor cells at a much lower concentration relative to normal cells. Further experiments to verify this hypothesis are warranted.

The anti-inflammatory effects of verapamil have been reported by several investigators (14, 15, 27, 28). For example in the study by Matsomuri, et al. (14) verapamil reduced the production of inflammatory cytokines (IFN-γ, TNFα and IL-1β) in PBMCs and in the study by Das, et al. (27) verapamil suppressed the recall of peritoneal macrophages in response to tioglicate. A verapamil induced decrease in the serum levels of TNF-α and IFN-γ in septic shock mice has been also reported (28). As gelatinases play an important role in inflammation (6), the anti-inflammatory properties of verapamil may be in part due to its inhibitory effects on gelatinase activity. The inhibitory effects of verapamil on LPS-induced secretion of pro-inflammatory cytokines and NF-Kappa B activation has been shown *in vivo* (14, 15). Suppression of the LPS-induced secretion of pro-inflammatory factors such as superoxide and nitric oxide (NO) by verapamil through a calcium channel- independent pathway has been shown (13). Inflammatory factors such as cytokines and prostaglandin E (2) are important regulators of MMPs activity (29, 30). Thus inhibitory effect of verapamil on MMP activity shown in this study may in part be due to its suppressive effect on inflammatory mediators.

The potential implication of anti-MMPs in the treatment of ischemic heart failure has been reported (31) and the positive effect of verapamil on ischemic heart failure (32) may in part be due to its anti- MMP activity. It should be noted that in our study, the concentration of verapamil which inhibited gelatinase activity *in vitro*, was higher than that usually used in cardiovascular patients.

In our previous study, verapamil showed a significant cytotoxic effect against human PBMCs at ≥1000 μM concentrations (33). Therefore a decrease in gelatinase activity at a verapamil concentration of 200 μM is not due to its cytotoxic effect and other mechanism(s) may be involved in this process.

Yue et al. (34) in a study of calcium channel blockers, showed that nifedipine increased and amlodipine decreased the MMP-2 activity in rat fibroblasts, while verapamil and diltiazem had no effect on MMP activity. The difference between our results and the Yue, et al. study may in part be due to differences between the lines and origin of the cells used. As already discussed, we used human PBMCs. However, the study by Yue, et al. used rat fibroblasts and another study performed by Yue et al. (35) in which verapamil had no effect on MMP-2 activity, the cells used were rat vascular smooth muscle cells.

According to our review of the literature this is the first report documenting the inhibitory effect of verapamil on gelatinase activity in human PBMCs. As gelatinases are important mediators of cancer and inflammation (6, 36), the anti-tumoral and anti-inflammatory effects of verapamil reported by other studies (12, 13) may in part be due to its inhibitory effects on gelatinase activity.

Given the important role of inflammation in cardiovascular diseases (37) the therapeutic effect of verapamil in cardiac disease (32) may in part be due to its anti-inflammatory properties mediated by inhibition of MMP(s)-activity.

Taken together our results suggest that verapamil, along with its long-term usage in cardiovascular diseases, may have potential implication for the development of MMP-inhibitors. Further studies of the effect of verapamil on gelatinase activity in other cell lines as well as inflammatory and autoimmune diseases are warranted.

## Conclusion

In this study, verapamil showed inhibitory effects on gelatinase (MMP-2/MMP-9) activity in human PBMCs. Thus verapamil may be of potential use in the preparation of MMP-inhibitors. MMPs have important role in inflammation, so verapamil, along with its long-term usage in cardiovascular disease, may be a good candidate for the development of anti- inflammatory agents.
